# Case report: a premature infant with severe intrauterine growth restriction, adrenal insufficiency, and inflammatory diarrhea: a genetically confirmed case of MIRAGE syndrome

**DOI:** 10.3389/fendo.2023.1242387

**Published:** 2023-09-06

**Authors:** Anna Go, Beom Hee Lee, Jin-Ho Choi, Jiyoon Jeong, Euiseok Jung, Byong Sop Lee

**Affiliations:** Department of Pediatrics, Asan Medical Center, University of Ulsan College of Medicine, Seoul, Republic of Korea

**Keywords:** MIRAGE syndrome, adrenal insufficiency (AI), enteropathy, hypoplasia, SAMD9

## Abstract

**Introduction:**

MIRAGE syndrome is a rare disease characterized by myelodysplasia, infection, growth restriction, adrenal hypoplasia, genital phenotypes, and enteropathy. Herein, we report the case of a girl with MIRAGE syndrome who presented with adrenal insufficiency and chronic diarrhea.

**Case presentation:**

The patient was born at 29 + 6 weeks of gestational age with a birth weight of 656 g (<3p). Her height and head circumference were also <3p. At birth, she presented with respiratory distress, meconium staining, and pneumomediastinum, which were managed with high-frequency ventilation and empirical antibiotics. Physical examination showed generalized hyperpigmentation and normal female genitalia. A few days after birth, polyuria and hypotension developed, and laboratory findings revealed hypoglycemia, hyponatremia, and hyperkalemia. Plasma adrenocorticotropic hormone levels were elevated with low serum cortisol levels and high plasma renin activity, which were suggestive of adrenal insufficiency. Hydrocortisone and fludrocortisone were introduced and maintained, and hyperpigmentation attenuated with time. Both kidneys looked dysplastic, and adrenal glands could not be traced on abdominal ultrasound. From the early days of life, thrombocytopenia and anemia were detected, but not to life-threatening level and slowly recovered up to the normal range. Despite aggressive nutritional support, weight gain and growth spurt were severely retarded during the hospital stay. Additionally, after introducing enteral feeding, she experienced severe diarrhea and subsequent perineal skin rashes and ulcerations. Fecal calprotectin level was highly elevated; however, a small bowel biopsy resulted in non-specific submucosal congestion. The patient was diagnosed with MIRAGE syndrome with SAMD9 gene mutation. She was discharged with tube feeding and elemental formula feeding continued, but chronic diarrhea persisted. By the time of the last follow-up at 15 months of corrected age, she was fortunately not subjected to severe invasive infection and myelodysplastic syndrome. However, she was dependent on tube feeding and demonstrated a severe developmental delay equivalent to approximately 5–6 months of age.

**Conclusion:**

The early diagnosis of adrenal crisis and hormone replacement therapy can save the life of -patients with MIRAGE syndrome; however, chronic intractable diarrhea and growth and developmental delay continue to impede the patient’s well-being.

## 1 Introduction

MIRAGE syndrome is a recently characterized multisystem disorder featuring adrenal hypoplasia. Primary clinical manifestations include myelodysplasia, infections, growth restriction, adrenal insufficiency, genital phenotypes, and enteropathy (1). MIRAGE syndrome is caused by a functional mutation in the *SAMD9* gene located on the long arm of chromosome 7 (7q21.2) (1, 2). This mutation modifies the endosome system, which serves as a negative regulator of cell proliferation and leads to hypoplasia of various organs (1). The most significant feature of MIRAGE syndrome is primary adrenal insufficiency (PAI), which can be life-threatening without prompt diagnosis and early treatment after birth (3–5). MIRAGE syndrome has an exceedingly high mortality rate, with most patients dying in infancy and early childhood due to complications such as infections and bleeding. However, an increasing number of survivors have been reported due to early diagnosis and supportive care, including steroid hormone replacement. Early and aggressive nutritional intervention is thought to improve patient outcomes, though enteropathy, which presents with intractable diarrhea, is troublesome and difficult to control.

This report presents the case of a preterm-born infant survivor diagnosed with MIRAGE syndrome, emphasizing the key characteristics of adrenal insufficiency and enteropathy.

## 2 Case presentation

The patient was delivered at 29 + 6 weeks of gestation due to oligohydramnios and fetal deceleration. The preterm-born infant had a birth weight of 656 g (-1.83SD), body length of 32 cm (-2.46SD), and head circumference of 22 cm (-3.35SD). Antenatal ultrasonography revealed intrauterine growth restriction, lung hypoplasia, pericardial effusion, and bilateral renal hypoplasia. The infant was born with severe respiratory distress, meconium staining, and pneumomediastinum. High-frequency ventilation was initiated, and empirical antibiotics were prescribed considering the sepsis concern.

**Figure 1:**
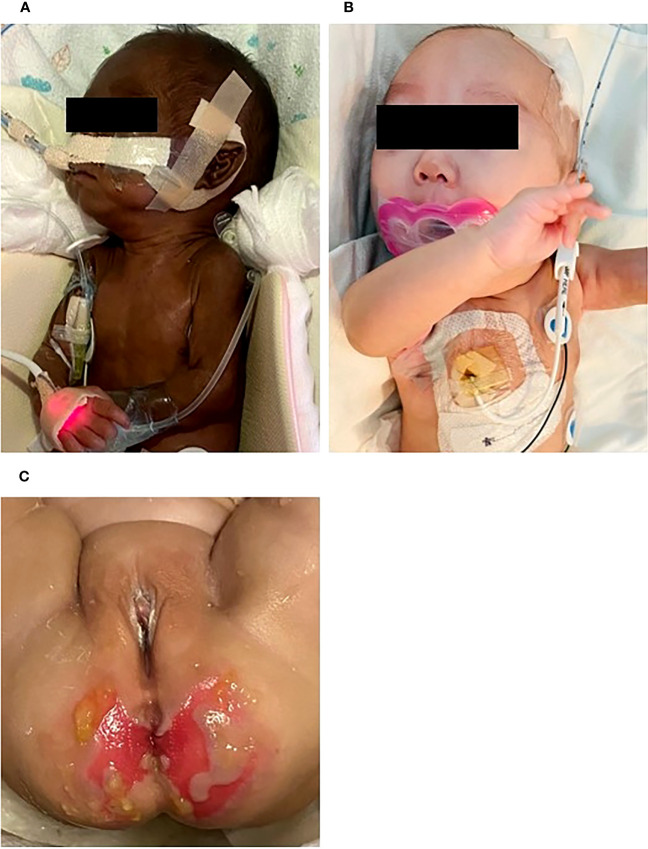
General appearance at **(A)** birth, **(B)** 5 months of postmenstrual age. At birth, there was notable hyperpigmentation, but over time, her skin color improved. She showed normal female genitalia. Perineal skin rashes and ulcerations developed due to chronic diarrhea at 4 months **(C)**.

Physical examination showed generalized hyperpigmentation and normal female genitalia (**Figure 1**). The infant developed polyuria (4-8 mL/kg/d) and hypotension a few days after birth. Further, the laboratory tests identified hypoglycemia, hyponatremia, hyperkalemia, anemia, and thrombocytopenia. Due to these clinical outcomes, adrenal insufficiency was highly suspected, which was ascertained by confirmatory tests.

Plasma adrenocorticotropic hormone (ACTH) levels were markedly elevated to 759 pg/mL (normal: 10-60 pg/mL), while plasma cortisol levels were significantly decreased to 1.3 μg/dL (normal: 2.5-9.1 μg/dL). Additionally, plasma renin activity was elevated at 36.4 ng/mL/h (normal: <2.8 ng/mL/h), and plasma aldosterone level was reduced to 5.1 pg/mL (normal: 190-1410 pg/mL). Plasma sodium was within the normal range at 137 mmol/L (normal: 135-145 mmol/L), while plasma potassium was increased to 5.7 mmol/L (normal: 3.5-5.1 mmol/L), indicating severe adrenal crisis. Imaging studies confirmed a dysplastic kidney and an invisible adrenal gland. Whole-exome analysis was performed using Sanger sequencing targeting the genetic disorders characterized by primary adrenal insufficiency and myelodysplasia, leading to the identification of a heterozygous *de novo* mutation in the *SAMD9* gene [NM_017654.4:c.2483C>G, (p.Pro828Arg)] and MIRAGE syndrome, approximately 1 month after birth. Hydrocortisone and fludrocortisone were initiated on the thirteenth day of life to treat the adrenal insufficiency and associated electrolyte imbalances. From day 21 in the hospital, hydrocortisone and fludrocortisone dosages were gradually increased, and the urine volume and potassium levels were slowly adjusted to the normal range.

**Figure 2:**
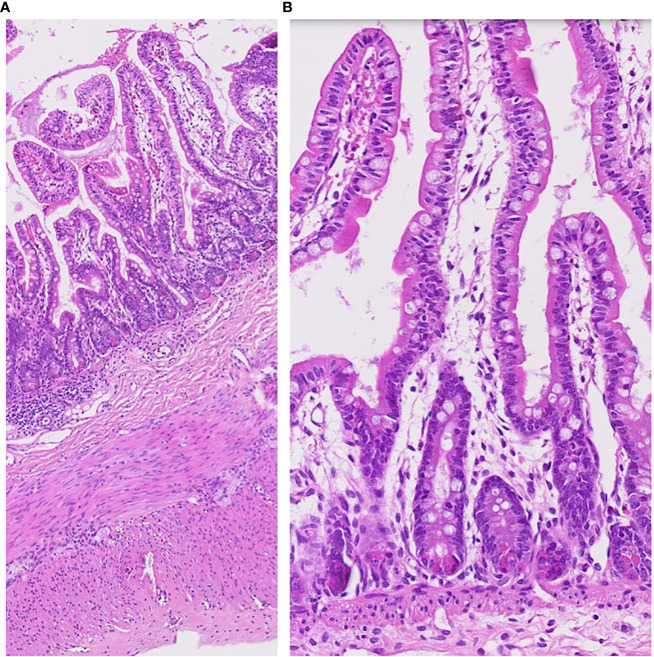
Pathology of small bowel biopsy at 18 weeks of her age. The small intestinal biopsy (jejunum) shows rather intact mucosal architecture with minimal inflammatory reaction **(A)**. Microvillus inclusions were not found **(B)**.

Special attention was paid to the nutritional supplementation of the preterm infant. She achieved full enteral nutrition with her mother’s milk and/or preterm formula at 2 weeks of age. We gradually increased her caloric intake with protein and non-protein fortified preterm formula (100 kcal/100 mL). At 10 weeks, the infant experienced loose stool, which changed to watery diarrhea. Despite including hypoallergenic formula and anti-diarrheal medication (Racecadotril), diarrhea was not improved. Over time, the buttocks became severely irritated, leading to frequent diaper rash and skin ulcerations (**Figure 1C**). We conducted serial fecal calprotectin tests to identify the etiology of intractable diarrhea in MIRAGE syndrome. An elevated calprotectin level (2027 μg/mg) was reported during the third week of the onset of diarrhea. After introducing an amino acid-based formula (Neocate^®^) at 14 weeks, the loose stool did not completely resolve, although there was some improvement in stool consistency, causing the healing of the severe perinatal diaper rash. The fecal calprotectin levels stabilized at 352 μg/mg after introducing Neocate^®^. Unexpectedly, hematochezia developed at 16 weeks of age and bowel ultrasonography revealed the signs of pneumatosis intestinalis, which highly suggested the occurrence of necrotizing enterocolitis (NEC) in preterm infants. She was managed with empirical antibiotics and nil per os (NPO) for approximately 2 weeks. The fecal calprotectin test did not significantly increase during this phase, measuring at 207 μg/mg. Inflammatory markers, including CRP, remained within the normal range, making the NEC diagnosis questionable. A small bowel biopsy was performed during inguinal hernia surgery 18 weeks after birth, which revealed non-specific findings, such as submucosal congestion, the normal density of submucosal and myenteric ganglion cells, and intact mucosal epithelia. Additional ultrastructural examination revealed intact mucosal epithelia without microvillus inclusions (**Figure 2**). During the birth admission, she had difficulty achieving full oral feeding. The infant also experienced challenges transitioning to complete oral feeding and remained on partial nasogastric tube feeding until the last visit at approximately 2 years of age.

Immediately after birth, transient thrombocytopenia was detected, slowly improving to a nearly normal range without dropping to a level that caused bleeding signs or required platelet transfusion. Regular hematologic check-ups for possible myelodysplasia through bone marrow biopsy showed no signs of bone marrow suppression. At 15 months, the platelet count was 126,000 per μL, hemoglobin was 13.0 g/dL, and white blood cell count was 12,500 per μL.

From birth to 19 months of age, she developed four episodes of serious infections requiring hospitalization: aspiration pneumonia (6 months of age), pneumonia (8 months of age), *Klebsiella pneumonia* sepsis (8 months of age), and urinary tract infection (8 months of age). The lymphocyte subset test showed a normal lymphocyte fraction. During every infection, hormone levels were monitored, and the hydrocortisone and fludrocortisone dosages were adjusted considering an adrenal crisis possibility.

**Figure 3:**
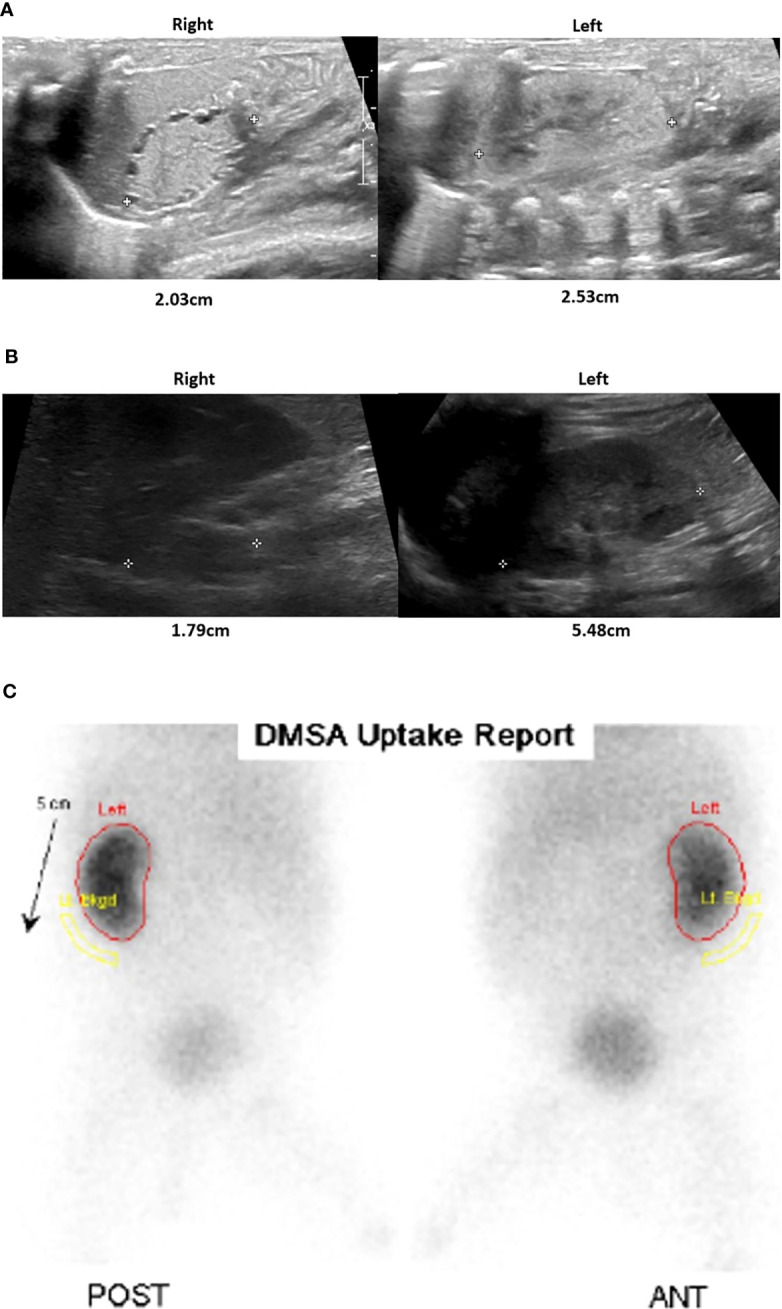
Renal and adrenal ultrasonography and renal scan. After birth, the parenchymal echogenicity of both the kidneys was increased, showing an undifferentiated corticomedullary junction and tiny cortical cysts. The right kidney was smaller than the left kidney **(A)**. At 20 months of age, there was a small right kidney, but the left kidney growth was relatively preserved, although cortical echogenicity remains increased **(B)**. In the Tc-99m Dimercaptosuccinic acid (DMSA) scan obtained at 6 months, the right kidney uptake is barely visible, indicating nonfunction, while the left kidney function appears unremarkable **(C)**.

In addition to adrenal hypoplasia, a renal ultrasound revealed bilateral dysplastic kidneys with a very small right kidney. While the left kidney demonstrated functional recovery with interval growth, the right kidney remained dysplastic and nonfunctioning (**Figure 3**). Elevated creatinine levels were observed initially, which were normalized within one month after birth. There was no laboratory finding suggesting renal tubular acidosis. In urinalysis, neither proteinuria nor hematuria was detected.

Brain MRI performed at approximately 4 months of age revealed no specific findings other than a probable cystic change of previous germinal matrix hemorrhage (GMH) grade I and an area of increased signal intensity on T2-weighted images in the left basal ganglia. At the chronological age (CA) of 6 months, a video fluoroscopic barium swallow study was conducted due to difficulties in oral feeding and frequent episodes of aspiration. The study revealed the presence of pharyngeal dysfunction, manifested by mild impairment in laryngeal protection and pharyngeal muscle weakness. Consequently, the infant was diagnosed with moderate to severe dysphagia and continued to depend on partial gastric tube feeding, as observed during the last follow-up at 20 months of CA.

In the Denver Developmental Screening Test conducted at a CA of 5 months, the infant showed a developmental delay of 3 months in all areas except for normal language development. However, the outpatient medical records evaluation at 20 months of CA showed significant developmental delays in all areas. The motor development level was around 8 months, while the cognitive/language development level remained at approximately 6 months. Rehabilitation therapy is ongoing for developmental delay. During the last outpatient clinic visit at 20 months of CA, the infant’s weight was 1 SD, height was -13.2 SD, and head circumference was -10.2 SD.

## 3 Discussion

In the present case, we performed genetic testing to determine the cause of adrenal insufficiency 1 week after birth. Early administration of hydrocortisone and fludrocortisone, even before genetic confirmation, was able to correct hypotension, hypoglycemia, polyuria, and electrolyte imbalances. Clinical interrogation and early intervention are crucial in preventing serious complications and improving the long-term treatment outcome for patients with primary adrenal insufficiency (6). The patient is relatively stable while maintaining hormone replacement therapy, though she shows cushingoid features. Monitoring the long-term effects of steroid treatment on growth and development is crucial, especially in premature infants.

In MIRAGE syndrome, the frequency of enteropathy is approximately 80%. The main enteropathy symptoms include chronic watery diarrhea and esophageal achalasia (7, 8). These can significantly impact affected individuals’ quality of life and overall prognosis. However, the underlying mechanism and pathology of enteropathy in MIRAGE syndrome remains unclear. Our patient suffered from feeding intolerance and chronic diarrhea beginning at 3 months of age, lasting for several months. To the best of our knowledge, this is the first case report describing the serial change in fecal calprotectin levels and findings of intestinal biopsy in MIRAGE syndrome.

**Figure 4:**
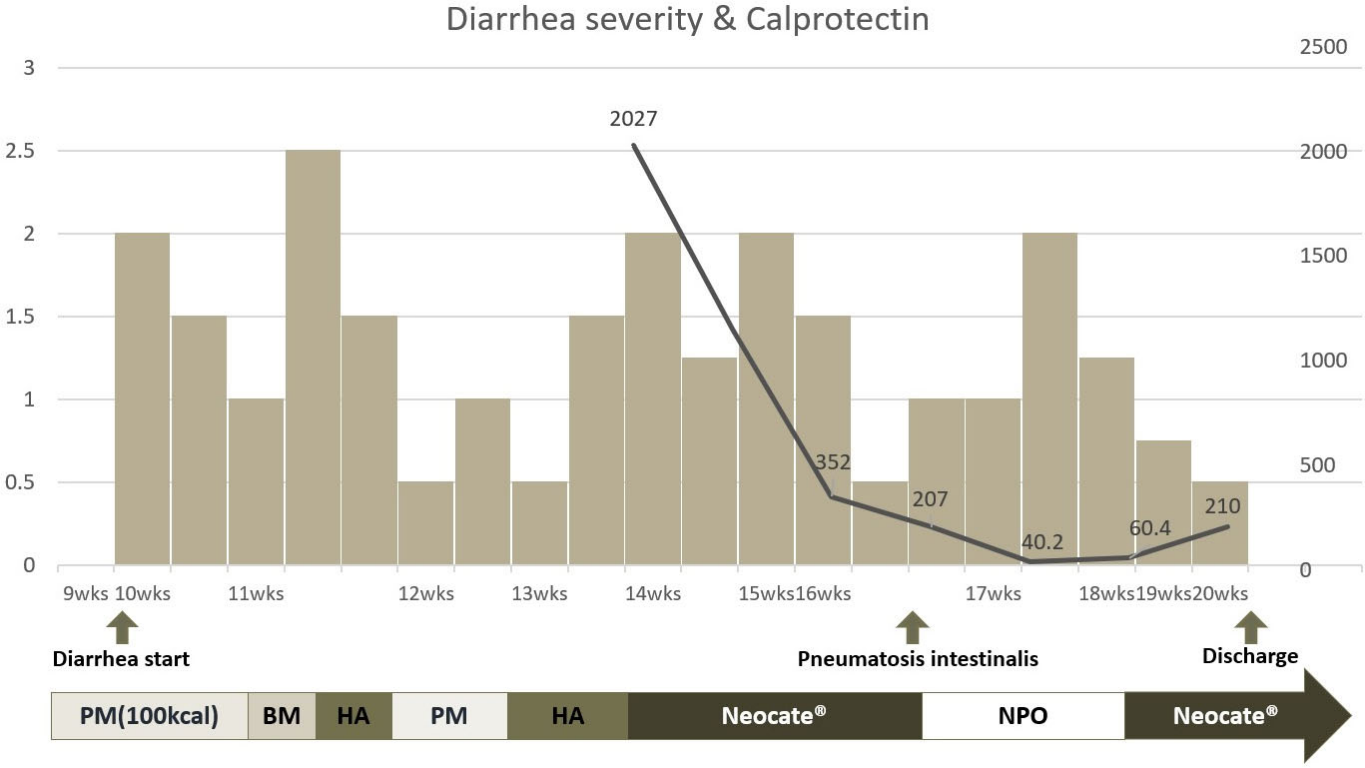
Clinical course of enteropathy and correlation of fecal calprotectin level and formula change. * HA, Hydrolyzed formula; BM, Breast milk; PM, Premature milk; Neocate^®^, amino acid based fully hydrolyzed formula; NPO, Nil Per Os. The left y axis of the graph is scored from 0 to 2.5 based on the Diapered Infant Stool Scale. The right y axis represents the fecal calprotectin level. The x-axis represents the period after infant birth. Additionally, a graphical representation was created to examine the correlation between calprotectin levels and formula changes.

In this case, we repeatedly measured fecal calprotectin to determine potential causes of severe, intractable diarrhea. Fecal calprotectin, a protein abundant in neutrophils and monocytes, is a valuable biomarker to assess inflammation in the intestinal mucosa (9, 10). The fecal calprotectin levels in neonates, including preterm infants, show an initial rise until the 7th day after birth, followed by a gradual decline over the next 6 weeks, with median values of approximately 132 μg/g (11, 12). In this case, fecal calprotectin level was high in the initial measurement at around 14 weeks post-birth but showed a decreasing trend after switching from a hydrolyzed formula to an amino acid-based, fully hydrolyzed, lactose-free formula. To assess the diarrhea severity, we modified the ‘Diapered Infant Stool Scale’ by implementing a 5-step scoring system, ranging from 0 to 2.5 (13). Using this score, we could correlate the severity of diarrhea with the corresponding fecal calprotectin levels. However, the severity of enteropathy did not correlate with changes in fecal calprotectin levels (**Figure 4**). Considering the characteristics of diarrhea worsening during the NPO period, a possibility of secretory diarrhea was anticipated rather than malabsorptive diarrhea.

Regarding early-onset neonatal diarrhea, two categories have been suggested: constitutional intestinal epithelial disorders and immune-inflammatory disorders (14). Pathologically, the former includes microvillus atrophy or epithelial dysplasia, while the latter includes autoimmune enteropathy or inflammatory colitis. Based on the small bowel biopsy results, the possibility of constitutive intestinal epithelial disorders as the underlying pathophysiology of intractable diarrhea is less expected in MIRAGE syndrome (15–17). It is worth noting that the infant’s fecal calprotectin did not increase during the period of suspected NEC. It remains unclear whether this discrepancy can be attributed to the increase in steroid dosage to prevent the risk of adrenal crisis during NEC because an increase in fecal calprotectin is generally reported during NEC (18–21). Although pneumatosis intestinalis is frequently observed in NEC, it is not a pathognomic sign and can also be seen in other conditions, such as bowel ischemia, severe gastroenteritis, and carbohydrate intolerance (22). Therefore, we cannot undermine the possibility of a misdiagnosis of NEC in our case. This observation may present a challenge in accurately interpreting the association between calprotectin levels and the severity of diarrhea.

In our case study, we implemented an amino acid-based fully hydrolyzed formula for managing chronic diarrhea in the patient starting at 4 months. While loose stools persisted, their severity was reduced, highlighting the importance of utilizing special formulas to ensure adequate nutrition during chronic diarrhea. Parenteral nutrition is an alternative option for long-term management; however, its challenges and associated risks, such as infections, prompted us to prioritize enteral feeding to maximize nutritional support. Amino acid-based formulas are commonly recommended for enteropathy associated with lactose intolerance or food allergies. Nevertheless, their efficacy in treating diarrhea caused by other factors, such as Crohn’s disease, remains uncertain (23). A recent case report demonstrated promising results in managing enteropathy with secretory and osmotic diarrhea, as well as autoimmune enteropathy. The porcine-derived pancreatic enzyme supplementation to the amino acid-based formula significantly improved the enteropathy symptoms. These findings suggest the potential benefits of adjunctive therapies in exploring the underlying mechanisms specific to diarrhea (24).

MIRAGE syndrome is associated with a reported average age of death at 3 years old, with a significant proportion attributed to infectious diseases (1, 2). In our case, the infant experienced recurrent aspiration pneumonia, otitis media, and one episode of sepsis; however, none progressed to life-threatening conditions. Furthermore, investigations were conducted to explore the underlying causes of recurrent aspiration pneumonia, but no concurrent gastroesophageal reflux or esophageal stricture was identified.

Growth retardation, in our case study, is presumed to be caused by intrauterine growth retardation and enteropathy associated with a heterozygous mutation in *SAMD9*. *SAMD9* plays a critical role in developing multiple organ systems and acts as a facilitator of endosome fusion. Mutations in *SAMD9* can lead to profound growth retardation (1). In summery, *SAMD9* plays a crucial role in growth and organ development, emphasizing the need for personalized care in MIRAGE syndrome.

This case report has notable strengths in elucidating the pathophysiology of enteropathy by tracking calprotectin levels and small bowel biopsies, which excluded constitutive intestinal epithelial disorders like enteropathy-associated epithelial dysplasia (ED). Another strength of this report is serial renal imaging studies that revealed a progression of renal dysplasia, a novel finding not yet reported in MIRAGE syndrome, highlighting the need for screening and monitoring renal dysplasia. The limitation of our case report is the lack of baseline and planned protocol regarding fecal calprotectin. We could only monitor the calprotectin trend starting from the 14th-week post-birth, which was 3 weeks after the onset of diarrhea. Despite our presentation, the role of stool calprotectin level as a severity marker in MIRAGE syndrome-associated enteropathy remains uncertain.

## 4 Conclusion

MIRAGE syndrome-associated enteropathy presents significant challenges, and further research is necessary to unravel the underlying pathophysiology. Despite the limitations, this report sheds light on the potential benefits of early diagnosis, steroid therapy, and specialized formulas in managing diarrhea and bowel inflammation. This case report provides valuable insights into the pathophysiology of enteropathy in MIRAGE syndrome, using calprotectin and small bowel biopsies. Continued efforts in research and comprehensive care approaches are essential to improve the outcomes and quality of life of individuals with MIRAGE syndrome.

## Data availability statement

The datasets for this article are not publicly available due to concerns regarding participant/patient anonymity. Requests to access the datasets should be directed to the corresponding author.

## Ethics statement

Written informed consent was obtained from the minor(s)’ legal guardian/next of kin for the publication of any potentially identifiable images or data included in this article. Written informed consent was obtained from the minor(s)’ legal guardian/next of kin for the publication of this case report.

## Author contributions

AG collected data, drafted the initial manuscript, and critically reviewed and revised the manuscript. BL coordinated and supervised data collection and manuscript drafting and revised the manuscript for intellectual content. All authors listed have made a substantial, direct, and intellectual contribution to the work and approved it for publication.
